# An integrated radiomics nomogram based on conventional ultrasound improves discriminability between fibroadenoma and pure mucinous carcinoma in breast

**DOI:** 10.3389/fonc.2023.1170729

**Published:** 2023-06-22

**Authors:** Hui Wang, Hailing Zha, Yu Du, Cuiying Li, Jiulou Zhang, Xinhua Ye

**Affiliations:** ^1^ Department of Ultrasound, The First Affiliated Hospital of Nanjing Medical University, Nanjing, China; ^2^ Department of Radiology, The First Affiliated Hospital of Nanjing Medical University, Nanjing, China

**Keywords:** radiomics, nomogram, breast fibroadenoma, pure mucinous carcinoma, conventional ultrasound

## Abstract

**Objective:**

To evaluate the ability of integrated radiomics nomogram based on ultrasound images to distinguish between breast fibroadenoma (FA) and pure mucinous carcinoma (P-MC).

**Methods:**

One hundred seventy patients with FA or P-MC (120 in the training set and 50 in the test set) with definite pathological confirmation were retrospectively enrolled. Four hundred sixty-four radiomics features were extracted from conventional ultrasound (CUS) images, and radiomics score (Radscore) was constructed using the Least Absolute Shrinkage and Selection Operator (LASSO) algorithm. Different models were developed by a support vector machine (SVM), and the diagnostic performance of the different models was assessed and validated. A comparison of the receiver operating characteristic (ROC) curve, calibration curve, and decision curve analysis (DCA) was performed to evaluate the incremental value of the different models.

**Results:**

Finally, 11 radiomics features were selected, and then Radscore was developed based on them, which was higher in P-MC in both cohorts. In the test group, the clinic + CUS + radiomics (Clin + CUS + Radscore) model achieved a significantly higher area under the curve (AUC) value (AUC = 0.86, 95% CI, 0.733-0.942) when compared with the clinic + radiomics (Clin + Radscore) (AUC = 0.76, 95% CI, 0.618-0.869, *P* > 0.05), clinic + CUS (Clin + CUS) (AUC = 0.76, 95% CI, 0.618-0.869, *P*< 0.05), Clin (AUC = 0.74, 95% CI, 0.600-0.854, *P<* 0.05), and Radscore (AUC = 0.64, 95% CI, 0.492-0.771, *P*< 0.05) models, respectively. The calibration curve and DCA also suggested excellent clinical value of the combined nomogram.

**Conclusion:**

The combined Clin + CUS + Radscore model may help improve the differentiation of FA from P-MC.

## 1 Introduction

Breast cancer (BC) is the most common malignant tumor in women, accounting for 31% of all diagnosed malignancies (1). Mucinous carcinoma (MC) is a rare subtype of breast cancer, accounting for 1%–6% of primary breast cancer (2), and can be divided into pure MC (P-MC) and mixed MC (M-MC) according to the content of mucin (2, 3). The mucus component of a tumor greater than 90% is defined as P-MC, while that of M-MC is 50%–90% (4). M-MC has typical malignant tumor manifestations and is easy to be diagnosed, whereas P-MC is indistinguishable from fibroadenoma (FA), especially FA accompanied by myxoid changes (5).

FA is the most common benign solid tumor in adolescents and young women, originating from the epithelium and stroma of the terminal duct lobular unit (6). FA is often found incidentally on physical examination and presents as painless, palpable, and mobile masses (7). Its size varies, even exceeding 5 cm (8). Due to different histopathologic characteristics and components, approximately 40% of FA is accompanied by myxoid or edematous changes (9), which makes it harder to distinguish from P-MC. P-MC and FA require different therapeutic approaches, so preoperative diagnosis is crucial (10).

**Figure 1:**
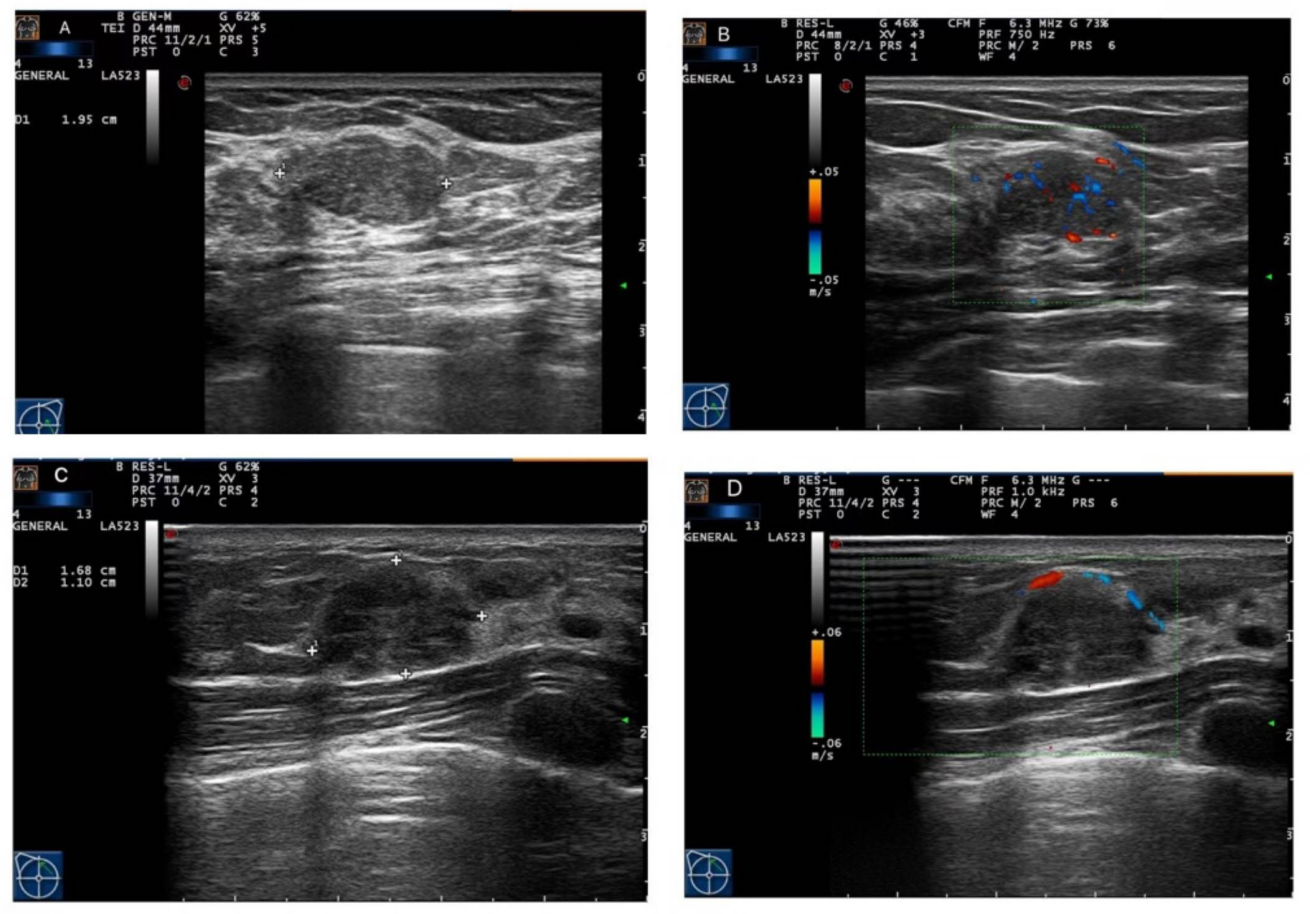
Ultrasound imaging of fibroadenoma (FA) and pure mucinous carcinoma (P-MC). **(A, B)** Images from a 46-year-old female patient. Ultrasound imaging showed that there was a hypoechoic nodule with a regular shape and circumscribed edge and rich blood flow in the left breast, which was confirmed as fibroadenoma by pathology. **(C, D)** Images from a 33-year-old female patient. Ultrasound imaging showed that there was a hypoechoic nodule with a regular shape and circumscribed edge and poor blood flow in the right breast, which was confirmed as pure mucinous carcinoma by pathology.

Conventional ultrasound (CUS) is most frequently used to distinguish between benign and malignant breast lesions in China because of its convenience, cost-effectiveness, and radiation-free feature (11). However, P-MC and FA share many radiographic characters in common, and it is difficult to distinguish them using CUS (**Figure 1**). Preoperative core needle biopsy (CNB) is an invasive examination and could not reflect the pathology of masses entirely for the heterogeneity of tumors. Otherwise, CNB may lead to complications such as severe bleeding and infection (12). Therefore, it is important to seek a non-invasive method to identify P-MC and FA and help clinicians make accurate clinical decisions (13, 14).

In recent years, as a new and exciting research field, radiomics has attracted more and more attention as it can be performed with most of the medical imaging methods, such as mammography, computed tomography (CT), magnetic resonance imaging (MRI), and ultrasound (15, 16). Radiomics is a quantitative analysis method converting imaging data into high-dimensional, mineable features, including characteristics that cannot be perceived by the naked eye, for improved decision support (17–20). Radiomics has been widely used in qualitative analysis, genetic analysis, efficacy evaluation, and prognosis prediction of various tumors (21, 22). However, as far as we know, there was no study utilizing CUS-based radiomics on the differentiation between breast FA and P-MC.

Therefore, the aim of this study was to evaluate the ability of the radiomics signature based on ultrasound imaging to distinguish between breast FA and P-MC.

## 2 Materials and methods

### 2.1 Patients

**Figure 2:**
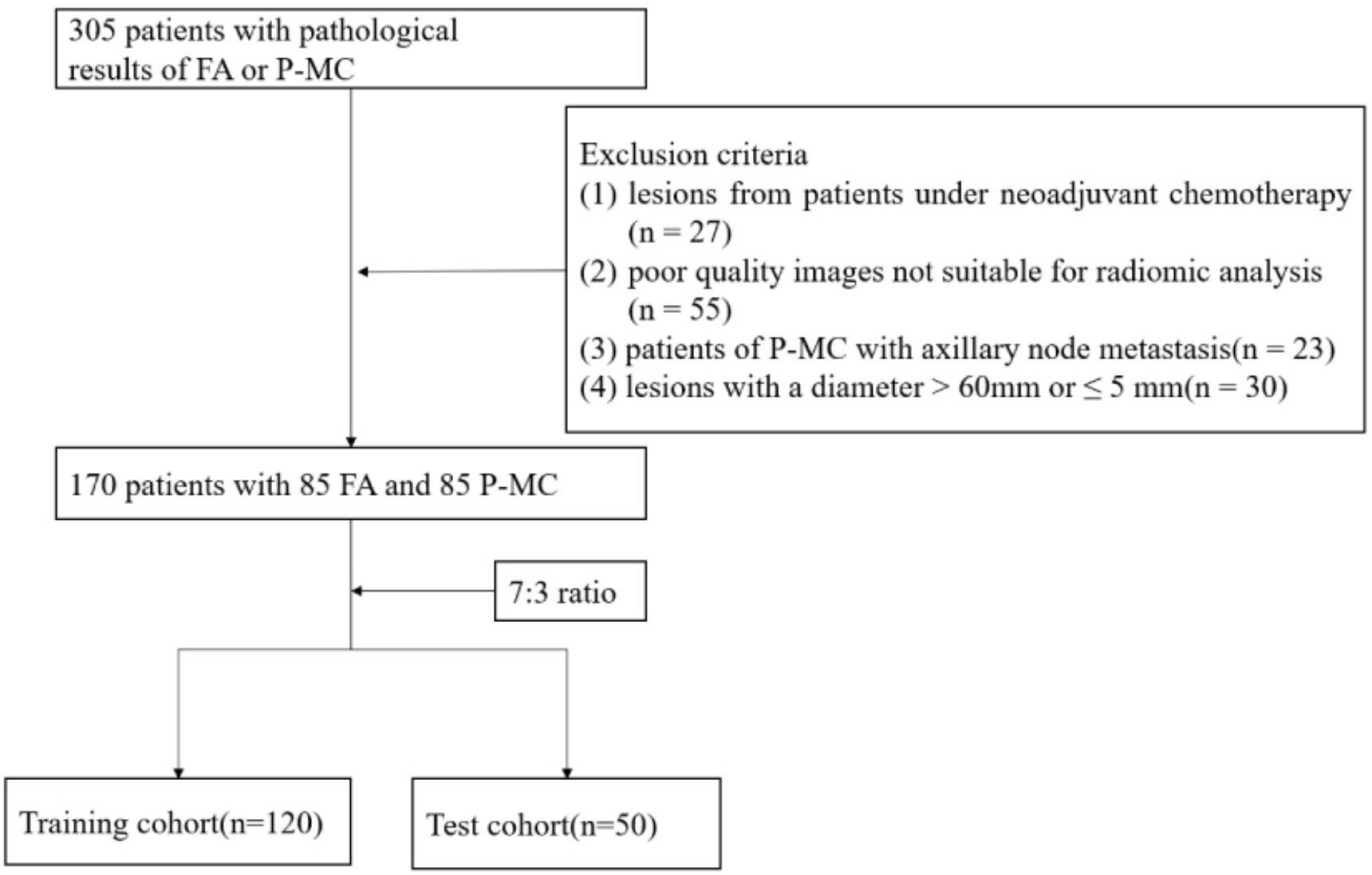
Flowchart of the patient enrollment.

Our Institutional Review Board approved this retrospective study and informed consent was waived. The study was conducted in accordance with the Declaration of Helsinki, and data from all patients were retrieved from the Picture Archiving and Communication System (PACS). From December 2014 to April 2020, a total of 305 patients with FA or P-MC with pathologic results were enrolled. The exclusion criteria were as follows: 1) lesions from patients under neoadjuvant chemotherapy, 2) poor quality images not suitable for radiomics analysis, 3) patients of P-MC with axillary node metastasis, and 4) lesions with a diameter >60 or ≤5 mm. Finally, 170 patients, consisting of 85 FAs and 85 P-MCs, were enrolled in this study and separated into a training cohort with 120 lesions (60 benign and 60 malignant lesions) and a test cohort with 50 lesions (25 benign and 25 malignant lesions) randomly with the ratio of 7:3 (**Figure 2**). Age was recorded as a basic clinical feature (**Table 1**).

**Table 1:** Comparison of the descriptive characteristics of the training and test cohorts.

Characteristic	Training cohort (*n* = 120)	Test cohort (*n* = 50)	*P*-value
Age (years)^a^	48.30 ± 19.15	44.84 ± 15.95	0.261
US-measured maximum diameter	20.00 (15.00-27.00)	21.50 (13.00-31.25)	0.776
Tumor echo Hypoechoic Isoechoic Mix echoic	114 (95.0%) 1 (0.8%) 5 (4.2%)	49 (98.0%) 0 (0.0%) 1 (2.0%)	0.632
Shape Regular Irregular	60 (50.0%) 60 (50.0%)	22 (44.0%) 28 (56.0%)	0.476
Edge Circumscribed Not circumscribed	80 (66.7%) 40 (33.3%)	34 (68.0%) 16 (32.0%)	0.866
Orientation Parallel Not parallel	95 (79.2%) 25 (20.8%)	44 (88.0%) 6 (12.0%)	0.174
Calcification Yes No Adler blood flow grade 0 I II III	13 (10.8%) 107 (89.2%) 20 (16.7%) 39 (32.5%) 44 (36.7%) 17 (14.1%)	11 (22.0%) 39 (78.0%) 15 (30.0%) 13 (26.0%) 14 (28.0%) 8 (16.0%)	0.057 0.222
Posterior echo No Enhancement Attenuation	105 (87.5%) 11 (9.2%) 4 (5.1%)	46 (92.0%) 4 (8.0%) 0 (0.0%)	0.406

Unless otherwise noted, data are shown as the number of patients, with percentages in parentheses.

US, ultrasonography.

a Data are means ± standard deviations.

### 2.2 Ultrasound image acquisition and interpretation

In order to avoid the discrepancies among different US machines, all of the images in this study were obtained by the same US machine with the same settings. All patients underwent CUS with a high-frequency linear transducer (LA523, 4–13 MHz) connected to an ultrasound system (MyLab Twice, Esaote, Italy). The patients were placed in a supine position to fully expose their breasts and armpits. Images of the largest transverse cross-section were routinely obtained for each lesion by two radiologists (reader 1 with 10 and reader 2 with 4 years of experience, respectively) who were blinded to the pathological results following the ACR BI-RADS fifth edition classification scheme. The ultrasonic characteristics comprised eight items: US-measured maximum diameter of the lesion, nodulous echo pattern, shape, margin, orientation, calcifications, Alder blood blow grade, and posterior echo (**Table 1**). If there were discrepancies, the consensus was reached after consultation. Logistic regression analysis was used to select the predictive clinical and ultrasonic factors of FA and P-MC in the training set, and the selected clinical and ultrasonic features (*P*< 0.05) were used to establish the Clin + CUS model by SVM.

### 2.3 Lesion segmentation and feature extraction

The region of interest (ROI) was segmented manually by a radiologist (reader 2) and confirmed by another experienced radiologist (reader 3 with 15 years of experience). Both radiologists were not aware of the clinicopathologic results. With the help of an open-source imaging program (ITK-SNAP), the ROI was delineated around the lesion outline. The feature extraction was implemented by the open-source Pyradiomics package (version 2.12; https://pyradiomics.readthedocs.io/en/2.1.2/). Then, a total of 464 radiomics features were retrieved as follows: a) first-order statistics features (*n* = 99) and b) texture features (*n* = 365). The first-order statistics only used the distribution of the values of the individual pixels without considering the spatial relationship. Texture features were used to describe statistical interrelationships between related pixels. To validate the repeatability of radiomics features, reader 2 and reader 3 delineated the ROIs on 50 random images, and reader 3 repeated the same procedure independently in a 1-week period. Finally, radiomics features whose intraclass correlation coefficient (ICC) and concordance correlation coefficient (CCC) values were greater than 0.8 were included in the subsequent analysis.

### 2.4 Radiomics feature selection and model development

**Figure 3:**
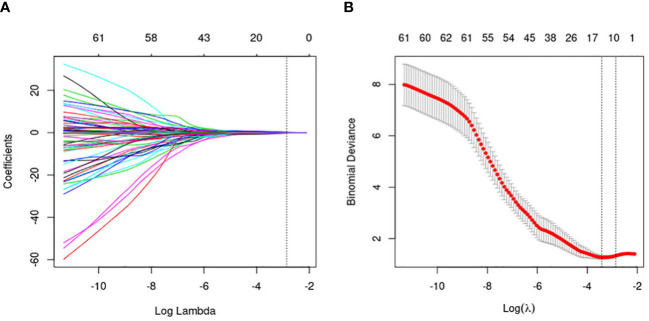
Flowchart of the radiomics feature selection. **(A)** The penalty value (*λ*) selection. Using 10-fold validation, the log(*λ*) was plotted based on 1 standard error of the minimum criteria. **(B)** LASSO coefficient profiles of the 464 radiomics features. Coefficient profiles were generated based on selected log(*λ*) values. Eleven radiomics features intersecting the line were finally included in the radiomics score, whose ordinate was the regression coefficient of the variable.

**Figure 4:**
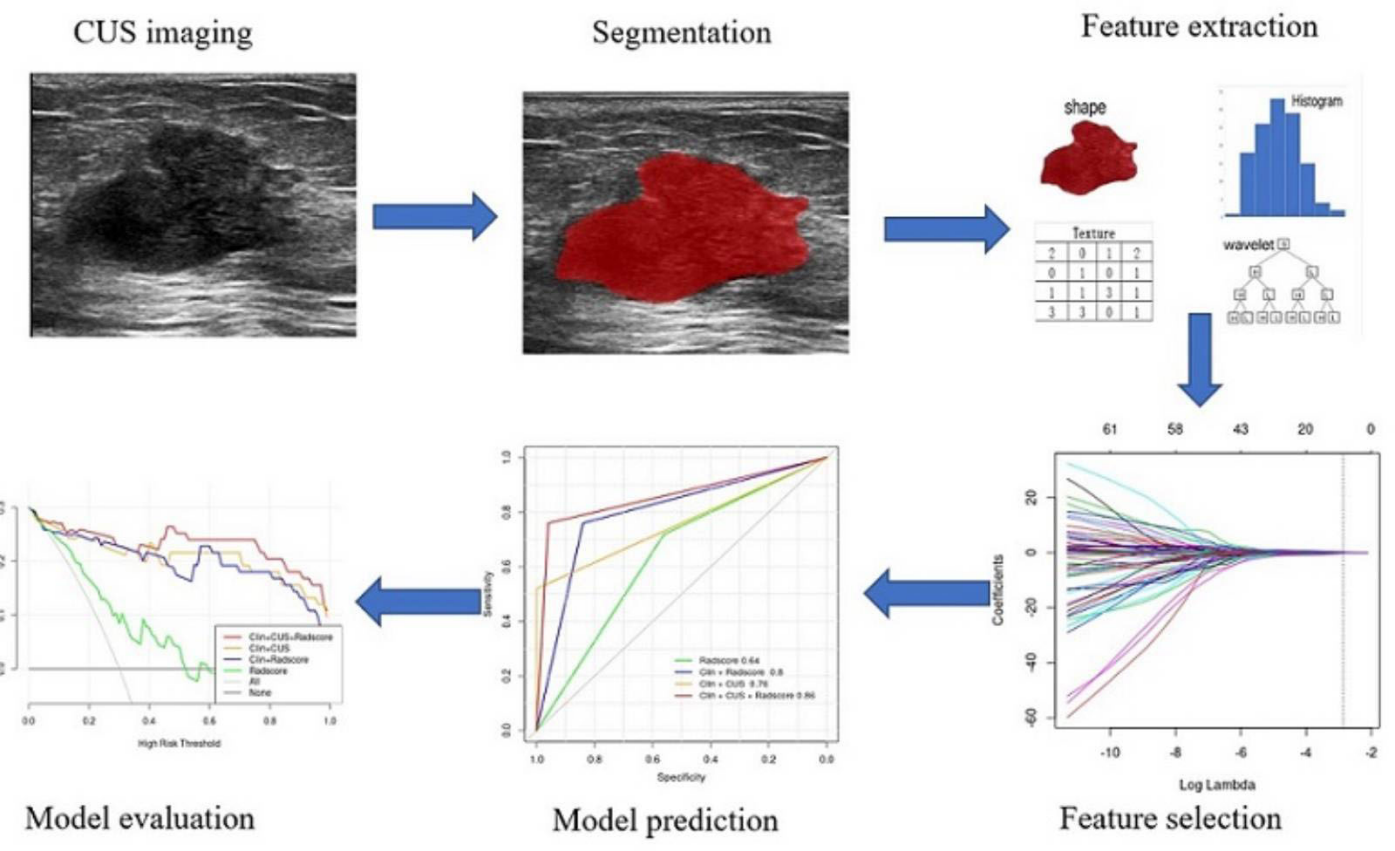
The flowchart of the radiomics analysis process.

Since the number of patients is less than the features, the model is prone to overfitting. So, to ensure the reliability of the model, the data are adopted to deal with dimension reduction. Least Absolute Shrinkage and Selection Operator (LASSO) is a machine learning algorithm used to filter and select radiomics features. The LASSO regression penalized parameters conducted by 10-fold cross-validation, making the coefficients of relatively unimportant characteristics return to zero (23). Finally, 11 features, which had a great influence on the discrimination between FA and P-MC, were selected, and then the selected features with their respective coefficients were used to build a radiomics signature, also known as Radscore (**Figure 3**). The flowchart of the radiomics analysis process is shown in **Figure 4**.

In view of the potential influence of statistically significant clinical characteristics and Radscore, different models were developed using SVM, including Clin + CUS, Clin + Radscore, and Clin + CUS + Radscore.

### 2.5 Statistical analysis

Data analysis was performed using the R software (version 3.6.2; http://www.R-project.org.). Continuous data were expressed as mean ± standard deviation, and categorical data as numbers or percentages. Differences in continuous data were compared using independent samples *t*-test, and categorical variables were compared using Fisher’s exact test or chi-square test. All tests were two-sided, and *P<*0.05 was considered statistically significant.

The performance of the different models was assessed based on their AUC values and compared using DeLong’s test. The calibration curves and the Hosmer–Lemeshow test were conducted to evaluate the differences between the predicted and observed data. To determine the clinical value of the different models in predicting FA and P-MC, decision curve analysis (DCA) was carried out by quantifying the net benefit under different threshold probabilities in the test cohort.

## 3 Results

### 3.1 Basic clinical information

There were 120 and 50 patients in the training set and test set, respectively. **Table 1** lists the clinical and CUS characteristics of the training and test cohorts, including age, tumor maximum diameter, tumor echo, shape, margin, orientation, calcification, Adler blood flow grade, and posterior echo. Between the training and test cohorts, basic clinical and CUS features were not statistically different (all *P* > 0.05).

### 3.2 Logistic regression analysis of clinical data and CUS features

In the training group, logistic regression was performed, and age, tumor maximum diameter, and orientation were considered independent predictive factors of identifying FA and P-MC (all *P*< 0.05). However, other CUS features were not independent predictors of FA and P-MC (*P* > 0.05) (**Table 2**). SVM was performed on the selected three features, and the Clin + CUS model was built.

**Table 2:** Logistic regression of clinical and ultrasonic features.

	Estimate	Std. error	*Z*	*P*
Intercept	−33.66369	1908.08551	-0.018	0.985924
Age	0.14761	0.04203	3.512	0.000445
Max_diameter	0.08743	0.04134	2.115	0.034445
Echo	14.21490	1908.08253	0.007	0.994056
Shape	0.65940	0.86466	0.763	0.445693
Edge	1.62137	0.95391	1.700	0.089187
Orientation	5.00813	1.52913	3.275	0.001056
Calcification	1.91927	1.18023	1.626	0.103911
Adler grade	−0.67314	0.44136	−1.525	0.127224
Posterior echo	0.70416	0.91080	0.773	0.439448

Max_diameter, maximum diameter; Std. error, standard error.

### 3.3 Radscore development

**Figure 5:**
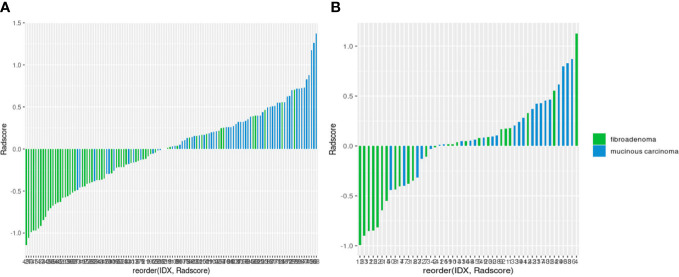
Waterfall plots of the radiomics signature-based classifier in the training **(A)** and test **(B)** sets.

Eleven radiomics features were selected from the CUS-based feature sets through LASSO analysis, consisting of two first-order statistics features and nine second-order features. The formula of Radscore generated with these selected features is as follows: $Radscore=z+\sum_{i=1}^{11}a_{i}*F_{i}$ (**Table 3**). As shown in the waterfall plot, Radscore had a good classification performance, and the higher the score, the greater the P-MC may be (**Figure 5**).

**Table 3:** Features in the radiomics score.

N_i_	a_i_	F_i_
1	2.140454e−01	original_shape2D_Elongation
2	−1.420049e−01	original_gldm_LowGrayLevelEmphasis
3	−9.419625e−02	wavelet.LH_glcm_Correlation
4	4.136180e−02	wavelet.HL_glszm_LargeAreaLowGrayLevelEmphasis
5	1.790443e−01	wavelet.HH_firstorder_RootMeanSquared
6	1.222509e−01	wavelet.HH_glszm_SizeZoneNonUniformity
7	−2.667396e−01	wavelet.HH_gldm_DependenceVariance
8	1.015937e−02	wavelet.HH_gldm_GrayLevelNonUniformity
9	1.311474e−01	wavelet.HH_ngtdm_Complexity
10	−2.096237e−01	wavelet.LL_glcm_DifferenceVariance
11	1.262036e−01	wavelet.LL_gldm_GrayLevelNonUniformity
*Z*	−4.236122e−05	

N_i_, serial number; a_i_, coefficient; F_i_, feature; Z, intercept.

### 3.4 Model establishment and validation

**Figure 6:**
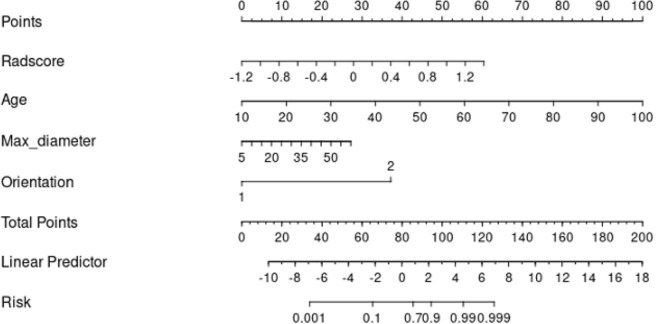
A nomogram combined with the clinical data, CUS features, and radiomics score to distinguish FA and P-MC in the training cohort.

**Figure 7:**
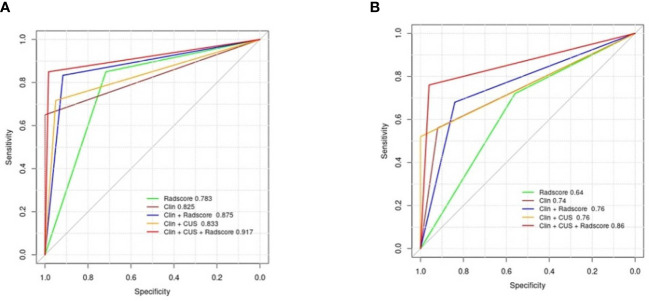
Diagnostic performance of the different models. **(A)** AUCs of the different models for predicting FA and P-MC in the training cohort; **(B)** AUCs of the different models for predicting FA and P-MC in the test cohort. Note: Clin, clinical mode; CUS, conventional ultrasound; Radscore, radiomics score; AUCs, the area under the receiver operating curves.

**Figure 8:**
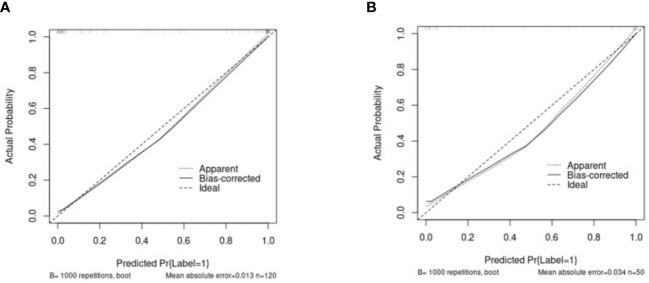
The calibration curves of the Clin + CUS + Radscore nomogram in the training **(A)** and test cohorts **(B)**. The *X*/*Y*-axes represent the predicted and actual risks of P-MC in the nomogram, respectively. The most ideal state is a solid line at 45°, meaning the prediction is exactly in line with the actual risk. When the dotted line is closer to the solid line, the consistency is better; otherwise, it is worse. Note: Clin, clinical mode; CUS, conventional ultrasound; Radscore, radiomics score.

The clinical data, CUS features, and radiomics score were combined as a nomogram by SVM in the training set (**Figure 6**). In the training and test cohorts, the AUC values of CUS + Clin + Radscore were 0.917 and 0.86, which were higher than those of the Clin + Radscore (AUC = 0.875 and 0.76, 95% CI, 0.802-0.928 and 0.618-0.869, *P* > 0.05), Clin + CUS (AUC = 0.833 and 0.76, 95% CI, 0.754-0.895 and 0.618-0.869, *P*< 0.05), Clin (AUC = 0.825 and 0.74, 95% CI, 0.745-0.888 and 0.600-0.854, *P*< 0.05), and Radscore (AUC = 0.783 and 0.64, 95% CI, 0.699-0.853 and 0.492-0.771, *P*< 0.05) (**Figures 7A, B**). Additionally, the specificity (96% *vs*. 84%) and the accuracy (96% *vs*. 80%) of this Clin + CUS + Radscore model were higher than those of the Clin + Radscore model (**Table 4**). The calibration curves suggested a high accuracy of the CUS + Clin + Radscore model for distinguishing FA and P-MC in the training and test sets (**Figure 8**). In the study, differences between predicted and observed data are confirmed as insignificant, with *P*-values of 0.2178 and 0.7282 in the Hosmer–Lemeshow test, indicating that the CUS + Clin + Radscore model has good calibration ability.

**Table 4:** Diagnostic performance of the different models in the training and test cohorts.

	AUC	Sen (100%)	Spe (100%)	Acc (100%)	NPV (100%)	PPV (100%)
Clin + CUS + Radscore						
Training Test	0.92 0.86	85.00 76.00	98.33 96.00	91.67 86.00	86.76 80.00	98.08 95.00
Clin + CUS Training	0.83	71.67	95.00	83.33	77.03	93.48
Test Clin + Radscore	0.76	52.00	100.00	76.00	67.57	100.00
Training Test	0.88 0.76	83.33 68.00	91.67 84.00	87.50 76.00	84.62 72.41	90.91 80.95
Radscore						
Training	0.78	85.00	71.67	78.33	82.69	75.00
Test	0.64	72.00	56.00	64.00	66.67	62.07
Clin						
Training	0.83	65.00	100.00	82.50	74.07	100.00
Test	0.74	56.00	92.00	74.00	67.65	87.50

Clin, clinical data; CUS, conventional ultrasound; Radscore, radiomics score; AUC, the area under the receiver operating characteristics; Sen, sensitivity; Spe, specificity; Acc, accuracy; PPV, positive predictive value; NPV, negative predictive value.

### 3.5 Clinical use

**Figure 9:**
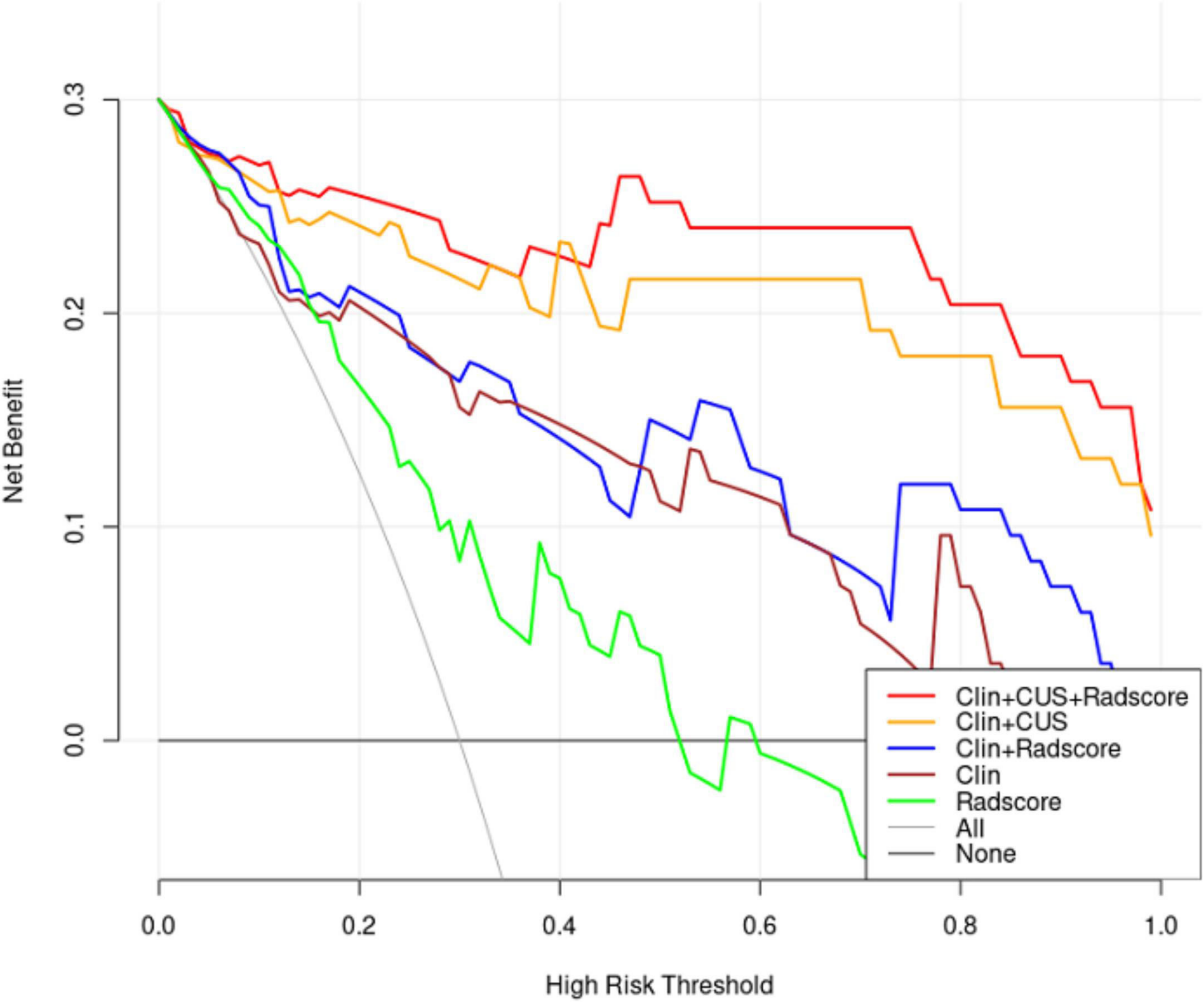
Decision curves of different models for predicting FA and P-MC in the test cohort. The curves revealed that applying the Clin + CUS + Radscore model would outperform than other models over a wide range of threshold probabilities. Note: All, treat all as P-MC; None, treat all as FA; Clin, clinical mode; CUS, conventional ultrasound; Radscore, radiomics score.

DCA was used as a means of estimating the clinical net benefit of the models (**Figure 9**). The results confirmed that the Clin + CUS, Clin + Radscore, and Clin + CUS + Radscore models possess excellent clinical value in distinguishing FA and P-MC at a wide range of risk threshold probabilities, and among these models, Clin + CUS + Radscore is the optimal.

## 4 Discussion

Our study showed that the radiomics nomogram, combining clinical ultrasound features with Radscore based on conventional ultrasound, performed excellently in distinguishing between FA and P-MC.

Pure mucinous carcinoma contains greater than 90% mucus and has closely analogous ultrasonic manifestation to breast benign tumor. P-MC is easily confused with FA, being difficult to distinguish by conventional ultrasound, and a few relevant studies were reported on their differential diagnosis. Our study found that age and tumor maximum diameter were independent predictors of P-MC and FA, which was in agreement with the study of Liang et al. (24). The older the patients are, the more likely they are to develop breast cancer (25, 26). Therefore, any new lesion that arises in the breast post-menopausal, even if it looks like a fibroadenoma, should be biopsied. This study found that the maximum diameter of P-MC was significantly larger than that of FA, which was consistent with previous studies (10, 27). According to the biological behavior of the tumor, malignant tumors increase rapidly in size, while benign tumors increase slowly or remain stable in size (24). In the present study, more than one-third of P-MC manifested as non-parallel, which is a feature of presumed malignant breast tumors (28). Considering CUS and clinical data comprehensively, Clin + CUS showed only moderate diagnostic performance in the test group (AUC = 0.76). Therefore, it is recommended to further improve the diagnostic performance of CUS.

A similar model—the multilayer perceptron (MLP) model—based on ultrasonic characteristics has been developed in a previous study, well distinguishing MC from FA, and the AUC was 0.919 (24). However, the MLP model only included conventional ultrasound features and did not extract and mine quantitative features from ultrasound images. Since the concept of radiomics was proposed by Lambin in 2012, radiomics has developed rapidly in the field of medicine (17, 29). Radiomics extracted a large number of quantitative features from digital images that cannot be distinguished by the naked eye, first applied in lung and neck tumor imaging and more recently in the field of breast ultrasound imaging (30–32). However, CUS-based radiomics has been validated for distinguishing benign and malignant breast tumors or predicting axillary lymph node metastasis (31, 33, 34). Whether radiomics plays a role in differentiating breast FA from P-MC has not been studied before. In this study, we established Radscore based on 11 features mined from CUS images, consisting of two first-order features and nine texture features. The first-order feature is used to perform some statistical analysis on the ROI of ultrasound images and obtain the corresponding statistics to describe the lesions at the gray level. Texture feature refers to a perceptible, measurable spatial change, viewed as a grayscale, a visual perception of local image features that can highlight details in the original image and quantify intratumor heterogeneity (11, 35). The radiomics feature that made the largest part in the Radscore is GLCM, which describes the distribution of two pixels having some kind of spatial relationship. This was consistent with previous research (36, 37).

To the best of our knowledge, this study is the first to develop a radiomics nomogram combining clinical data, CUS features, and radiomics score to distinguish FA and P-MC. In this study, Clin + CUS + Radscore showed excellent diagnostic performance (AUC = 0.86) when compared with Clin + Radscore (AUC = 0.76), Clin + CUS (AUC = 0.76), Clin (AUC = 0.74), and Radscore (AUC = 0.64), respectively. Additionally, the sensitivity and accuracy of this combined model also performed best among all models. The calibration curve showed that predictive probability was in high agreement with actual probability, signifying good stability with the radiomics nomogram. The DCA further confirmed that the Clin + CUS + Radscore model can improve the effectiveness of individual clinical decision-making, providing a novel approach to distinguish FA and P-MC non-invasively and accurately.

There were several limitations in this study. First, the sample size in our study was relatively small and involved single-center research; thus, multicenter studies with a large sample size are necessary. Second, this retrospective study may lead to selection bias. Third, although manual segmentation was used in this study, the ICC and CCC were good, and we believed that they had a little impact on the results. We will try to use automatic segmentation in our further research. Fourth, there were fewer variables in the clinical model, and we will add more clinical variables, such as blood serum indicators and family history, in our future study. Lastly, we only studied the radiomics signature based on CUS in this study. Multimodal ultrasound-based radiomics such as contrast-enhanced ultrasound and elastography may show better diagnostic performance.

In conclusion, our findings suggest that the Clin + CUS + Radscore model can effectively differentiate FA and P-MC, thus improving the confidence of radiologists as well as assisting in clinical decision-making. Further study is needed to validate our findings on a broader multicenter patient sample.

## Data availability statement

The original contributions presented in the study are included in the article/supplementary material. Further inquiries can be directed to the corresponding authors.

## Ethics statement

Written informed consent was not obtained from the individual(s) for the publication of any potentially identifiable images or data included in this article.

## Author contributions

HW: data curation, formal analysis, and writing—original draft. HZ: software and validation. YD: visualization and investigation. JZ: methodology and software. CL: conceptualization and supervision. XY: conceptualization and supervision. All authors contributed to the article and approved the submitted version.
